# Differential Methylation in Promoter Regions of the Genes *NR3C1* and *HSP90AA1*, Involved in the Regulation, and Bioavailability of Cortisol in Leukocytes of Women With Preeclampsia

**DOI:** 10.3389/fmed.2020.00206

**Published:** 2020-06-16

**Authors:** Quitzia Torres-Salazar, Yolanda Martínez-López, Miguel Reyes-Romero, Rebeca Pérez-Morales, Antonio Sifuentes-Álvarez, Jaime Salvador-Moysén

**Affiliations:** ^1^Instituto de Investigación Científica, Universidad Juárez del Estado de Durango, Durango, Mexico; ^2^Facultad de Medicina y Nutrición, Universidad Juárez del Estado de Durango, Durango, Mexico; ^3^Facultad de Ciencias Químicas Campus Gómez Palacio, Universidad Juárez del Estado de Durango, Durango, Mexico; ^4^Hospital Materno Infantil del Estado de Durango, Durango, Mexico

**Keywords:** preeclampsia, pregnancy, methylation, cortisol, hypothalamic–pituitary–adrenal axis

## Abstract

**Introduction:** Hypertensive disorders are of interest in obstetrics and gynecology because they are the second place among causes of maternal mortality and a source of complications in the short, mid, and long term. Even if the pathophysiological process behind preeclampsia (PE) is still unknown, stress factors have been revealed to play an important role in the genesis of this pathologic process.

**Methods:** A case-control study was designed with the purpose of determining if there is a differential methylation in *NR3C1, HSD11B2, CYP11A1, CRHBP, TEAD3*, and *HSP90AA1* genes, related to signaling of the hypothalamic–pituitary–adrenal axis, and its regulation on early-onset PE (EOPE).

**Results:** A total of 20 cases and 20 controls were studied by DNA methylation analysis, demonstrating differences among groups in the percentage of methylation of the *NR3C1* gene. After a contingency analysis, an odds ratio (OR) for PE of 12.25 was identified for *NR3C1* and 9.9 for *HSP90AA1* genes*. NR3C1, TEAD3*, and *HSP90AA1* genes showed a positive correlation with the systolic and diastolic blood pressure levels with a *p* ≤ 0.05.

**Conclusion:** This study found a differential methylation in the glucocorticoid receptor (GR) *NR3C1* and its co-chaperone *HSP90AA1* in women with PE, with a possible regulatory role in the response to stress in pregnancy and is a likely physiopathological mechanism in PE.

## Introduction

Preeclampsia (PE) is a multisystemic syndrome of pregnancy and the puerperium, with a reduction of systemic perfusion, generated by vasospasm and the activation of coagulation systems (1). Approximately 10–15% of maternity deaths are directly associated with PE, with an estimated toll of 75,000 maternal deaths and 500,000 neonatal deaths per year. In Mexico, it constitutes the main cause of death associated with complications during pregnancy and represents 34% of the total maternal mortality (2).

It has been proven that the risk of developing PE is related to the maternal psychosocial status during pregnancy, requiring the evaluation of family and marital and social conditions. A disproportionate response to stress results in a metabolic derailment in the regulation by the hypothalamic–pituitary–adrenal (HPA) axis (3). Likewise, maternal stress and/or a rise of glutocorticoids *in utero* is associated with low birth weight and metabolic disorders among the offsping, in adulthood (4). PE is a syndrome of idiopathic origin that can present manifestations in practically all apparatuses and systems of the fetus–maternal binomial. The causal framework of PE is still poorly understood, but the literature is inclined to explain it through the deficient processes of placentation (5).

Various studies have documented an association between adverse cultural and psychosocial situations with a high frequency of gestational morbimortality (6, 7). Research-based approaches establish a clear association between a context characterized by high social stressors and low psychosocial support with the expression of PE. Studies in the area of psychoneuroimmunoendocrinology have shown that individuals exposed to chronic stress, anxiety, and/or other factors undergo immunologic disorders, suggesting that stress and emotions interfere with immunoregulatory mechanisms (8). Glucocorticoids produced in the adrenal cortex, in response to the signals from the HPA axis, can affect different cellular functions during pregnancy. It has also been reported that in pregnant women with PE, a premature rise of placental cortisol, joined by a reduced activity of the enzyme 11-beta-hydroxyesteroid dehydrogenase type 2 (HSD11B2), transforms cortisol into cortisone. Glucocorticoids also inhibit the luteinizing hormone (LH), estrogens, progesterone, and the ovarian steroid hormones. Significantly, a high activity of glucocorticoids established by an increase in the levels of cortisol in circulation or the altered metabolism of cortisol can lead to a resistance to insulin, closely associated with hypertension and endothelial resistance—characteristics observed along with PE (9).

The association between PE and epigenetic alterations of the placenta (6–8) has been recently reported. The maternal epigenome has a unique history that can be useful to study in order to understand its susceptibility to develop PE, as well as to detect it in its early stages (10). Contrary to the genotype (non-modifiable), epigenetics can confer a degree of phenotypic plasticity linked to stress, nutrition, or any other change in the environment, allowing the fetus to respond to the environment and making changes in the genetic expresion in accordance to this. Furthermore, if the epigenetic alterations occur in the gametes, these can be inherited and can bring phenotypic consequences to the next generation; for example, the possibility of epigenetic disorders previous to conception or during pregnancy can increase the susceptibility to PE (10).

A study describes a different pathophysiological behavior in early-onset PE (EOPE) and in late-onset PE (LOPE). They informed, in a case-control study, that in EOPE, a differential percentage of methylated DNA in CpG sites in genes encode for the receptor of glucocorticoids (*NR3C1*) and binding protein to CRH (*CRHBP*) (11). In othes studies are described the association between the degree of the methylation of the promoters of the gene *NR3C1*, with the presence of normotension, hypotension, and hypertension in a group of pregnant women, and how these can be found present independently from confounding variables such as tobacco use, family history of High Blood Pressure (HBP), or body mass index (BMI) (12, 13).

The purpose of this work was to determine if there is a differential methylation in *NR3C1, HSD11B2, CYP11A1, CRHBP, TEAD3*, and *HSP90AA1* genes related to signaling of the HPA axis and its regulation on EOPE.

## Materials and Methods

### Subjects

The present study was revised and approved by the Commitee of Ethics in Research of the General Hospital of Durango with registration number 456/015.

A paired case-control study was designed. The cases were pospartum women with (1) EOPE diagnosis, (2) pregnancy between 20 and 34 weeks of gestation, (3) age between 18 and 45, and (4) residents of the State of Durango, México. The participants in the control group were pospartum women without any complications. The groups were paired individually by age, place of residence, and pregnancy stage.

The size of the sample was calculated with the aid of SigmaPlot software, considering a minimal methylation difference to detect 12% among the study groups, a power of 95%, and an α value of 0.05, resulting in the groups composed of 20 cases and 20 controls.

Patients excluded from the study were all women with comorbidities (hypertension, diabetes, rheumatoid arthritis, systemic lupus erythematosus, psychiatric illness, antiphospholipid syndrome, Down syndrome, ischemic cardiopathy, kidney diseases) and women who presented PE in previous pregnancies, multiple pregnancies, hydatidiform mole, and miscarriages.

We used a case report format for the recollection of sociodemographic data. The American College of Obstetricians and Gynecologists 2013 criteria for the diagnosis of PE were considered, which include (1) systolic blood pressure ≥140 mmHg and/or diastolic blood pressure ≥90 mmHg, (2) pregnancy of more than 20 weeks of gestation, and (3) ≥300 mg proteinuria in 24-h urine or ≥1+ in test strip (14).

### Measures and Samples

Blood pressure was determined by an OMRONHEM7121 sphygmomanometer, the gestation week was inferred by ultrasound and proteinuria by urine analyses. The 24-h urine sample was used for the determination of proteinuria; the sample was collected into a sterile vessel. In a maximum period of 24 h postpartum, peripheral blood samples (BD Vacutainer® EDTA 4 ml) were collected from each participant. All the samples were frozen until the moment of their analysis, for DNA purification utilizing the standardized procedure described by Iranpur et al. (15). The quantification of DNA (A 260 nm), as well as the determination of purity (A 260/280), was conducted through spectrophotometry in a DU-730 Uv-Vis Bekman Coulter spectrophotometer. The DNA samples were stored at −70°C until processing in methylation assays.

### DNA Methylation Analysis

An *in silico* study was carried out utilizing the online Eukaryotic Promoter Data Base (EDP) software to locate the CpG Islands in/or near the transcription start site of candidate genes (16). The methylation analysis was conucted *via* qPCR employing reagents EpiTectMethyl II qPCR Primer Assay and EpiTect II DNA Methylation Enzime Kit (Qiagen®) in accordance with the instructions of the manufacturer. The determination of the percentage of methylation with these reagents is based upon the digestion of the restriction sites with methylation-sensitive enzymes. The genomic coordinates of the analyzed CpG islands are presented in the Supplementary Table 1. The reactions of qPCR in real time were conducted in a thermal cycler ECO Illumina® (San Diego, CA) using the v.1 software. To transform the Ct values obtained from the reaction of a PCR to percentages of methylation, a spreadsheet provided by Qiagen® was employed.

### Statistical Analyses

The data were analyzed with the statistical program SPSS v.21, central tendency and dispersion measures were calculated. The *T*-test was used to compare means between groups, in cases where medians were compared, the Mann–Withney *U*-test was used. The chi square test was used to compare percentages. An analysis of the correlation of clinical variables with methylation percentage was carried out employing Spearman's rho. The cutoff points for the contingency analysis were performed with the online software Cutoff Finder (17), and the odds ratio (OR) was calculated for each of the genes in the study. A value of *p* ≤ 0.05 of statistical significance was considered.

## Results

Between October 2015 and October 2016, 830 postpartum women were screened for the study, out of which 112 presented a PE clinical condition, from whom 20 were selected by fulfilling the inclusion criteria. The sociodemographic and clinical variables of the participants are presented in Tables 1, 2.

**Table 1:** Sociodemographic characteristics from a sample of women diagnosed with preeclampsia and normotensive.

		**Cases (*****n*** **=** **20)**		**Controls (*****n*** **=** **20)**		***p***
**ORIGIN**						
	Rural	15	75.0%	15	75.0%	
	Urban	5	25.0%	5	25.0%	1
Occupation	Seamstress	1	5.0%	0	_	
	Teacher	1	5.0%	0	_	
	Housewife	17	85.0%	19	95.0%	
	Office employee	0	_	1	5.0%	
	Student	1	5.0%	0	_	0.391
**MARITAL STATUS**						
	Single	3	15.0%	7	35.0%	
	Married	6	30.0%	2	10.0%	
	Domestic partnership	11	55.0%	11	55.0%	0.87

**Table 2:** Clinical information obtained from a sample of preclamptic women vs. normotensive.

	**Cases (*****n*** **=** **20)**		**Controls (*****n*** **=** **20)**		***p***
Maternal age^*^	28.45	± 7.6	28.5	± 7.8	0.969
Gestational age^**^	31	(28−33)	39.3	(38−40)	0.001
SBP^**^	160	(150−172)	110	(92−120)	0.001
DBP^**^	105	(91−110)	67.5	(60−77)	0.001
MBP^**^	122	(112−127)	81.6	(73−89)	0.001
	***n***	**%**	***n***	**%**	
**Sex of baby**					
Male	12	60.0%	13	65.0%	
Female	8	40.0%	7	35.0%	0.114
**End of pregnancy**					
Cesarean section	19	95%	4	20.0%	
Vaginal delivery	1	5%	16	80.0%	0.001
First pregnancy	9	45.0%	3	15.0%	
More than one pregnancy	11	55.0%	17	85.0%	0.011

SBP, Systolic blood pressure; DBP, Diastolic blood pressure; MBP, Mean blood pressure.

* mean and standard deviation.

** *median and quartiles 25 and 75*.

The methylation analysis showed differences among the groups for *NR3C1* gene with a median in the case group of 89.21 (interquartile range 73.3–92.2) vs. 22.1 (0.27–50) in the controls (*p* = 0.003) (Table 3). The methylation percentage of the genes *TEAD3, HSP90AA1, CYP11A1, HSD11B2*, and *CRHBP* did not show any statistically significant differences; however, a marginal association can be perceived in the first two, which presented significance in posterior analyses (Table 3).

**Table 3:** Differential methylation in the CPG islands of gene regulators of hypothalamic-pituitary-adrenal axis.

**Gene**	**Chromosomal location**	**CpG Island**	**Mean of % of methylation (q25–q75)**				***P***
			**Women with preclampsia**		**Women without preeclampsia**		
*NR3C1*	5q31.3	111,914	89.2	(73.3–97.2)	22.1	(0.27–50)	0.0003^*^
*CYP11A1*	6p21.31	112,418	44.0	(0.8–88.4)	3.7	(0.42–68.7)	0.377
*TEAD3*	16q22.1	105,382	90.0	(50–97.2)	51.8	(1.72–81.6)	0.077
*HSD11B2*	15q24.1	104,679	60.7	(1.46–95.4)	80.8	(1.73–99.90)	0.446
*HSP90AA1*	14q32.31	104,327	70.2	(0.40–79.4)	1.3	(0.05–8.4)	0.068
*CRHBP*	5q13.3	111,616	50.0	(33.12–64.1)	40.0	(0.52–95.2)	0.579

N = 20 cases and 20 controls.

* *p < 0.05*.

The correlation analysis of clinical variables with the methylation percentage showed a direct proportional relation between the methylation and elevation of blood pressure for genes *NR3C1, TEAD3*, and *HSP90AA1*, particularly for diastolic pressure (Table 4).

**Table 4:** ^a^Correlation analisis between gene methylation and blood pressure.

	**SBP**		**DBP**	
**Gene**	**Coefficient of correlation**	***p***	**Coefficient of correlation**	***p***
*NR3C1*	0.547	0.002^*^	0.562	0.001^*^
*CYP11A1*	0.202	0.27	0.19	0.305
*TEAD3*	0.469	0.05^*^	0.551	0.018^*^
*HSD11B2*	−0.058	0.755	0.071	0.706
*HSP90AA1*	0.379	0.052	0.386	0.046^*^
*CRHBP*	0.131	0.525	0.222	0.275

a The information was analyzed using the Spearman's rank correlation.

N = 40.

* Statistical significance ≤ 0.05.

*SBP, Systolic blood pressure; DBP, Diastolic blood pressure*.

Finally, a contingency analysis was performed to determine the OR in the presence or absence of PE and the methylation state of each of the genes in the study; finding ORs of 12.25 and 9.9 for *NR3C1* and *HSP90AA1* genes, respectively, both statistically significant. The remaining genes (*CYP11A1, HSD11B2, CRHBP*) did not present an association with the developmet of PE (Table 5).

**Table 5:** Hipermethylation frequency^a^ in the genes of study.

	**Women with preeclampsia (%)**	**Women without preeclampsia (%)**	***x^**2**^***	***P***	**OR**	**95% CI**
*NR3C1*	46.7	6.7	6.13	0.013^*^	12.25	(1.27–118.37)
*CYP11A1*	56.2	40.0	0.819	0.366	1.92	(0.46–8.05)
*HSD11B2*	50.0	60.0	0.313	0.576	0.66	(0.16–2.77)
*HSP90AA1*	64.3	15.4	6.6	0.01^*^	9.9	(1.54–63.69)
*CRHBP*	15.4	30.8	0.352	0.867	0.4	(0.06–2.77)

a The cut point was taken as appointed by the cut off finder (18).

* Statistical significance p < 0.05.

*Differences between cases and controls*.

## Discussion and Conclusions

In this study, differences in the percentage of DNA leukocyte methylation in the study groups were demonstrated, particularly in that which refers to the *NR3C1* gene. This result is congruent with that reported by Hogg et al. (11) who described higher methylation in the placenta of women with EOPE when compared with healthy controls.

With reference to genes *CYP11A1, TEAD3, HSD11B2, HSP90AA1*, and *CRHBP*, when a clustering categorization is executed, we obtain significant differences in *NR3C1* and *HSP90AA1* genes, presenting 12.25 and 9.9 more probability of developing PE in those women in hypermethylation states in comparison with those who presented a lower methylation, with 95% CI that is statistically significant. The results are also consistent with that reported in 2017 by Dwi Putra et al. (12) who found an association between the regulation of blood pressure in pregnant women and the methylation of the promoter of *NR3C1*.

Even though there are no reports in the literature that directly relate methylation of this co-chaperone with PE, Ilona et al. (18) demonstrated an association between the expression of the heat shock protein HSP70 and pregnancy complications. In other HSP proteins, like 27, 60, and 90, no differences were found due to the small size of the sample. Our findigs agree with the theoretical foundation of the function of the co-chaperone HSP90, which promotes the maturation of the glucocorticoid receptor (GR), parting from an appropriate folding state in conformity to where it has a high cortisol affinity.

Only concluding data are presented in the differences found in *NR3C1* and *HSP90AA1*. Although a disparity was observed among the medians of other genes in the study (*CYP11A1, TEAD3*), this did not achieve a statistical significance of <0.05; however, we propose that they serve as a reference for future research, probably with a larger study sample size.

The GR exerts its function on the HPA axis, and several studies have demonstrated its expression in a wide diversity of cell types. Therefore, its regulation includes DNA methylation, alternative splicing, and translational mechanisms (19). This indicates that its regulation is very complex, and the expression-specific tissue depends on this process. However, methylation is a mechanism that has proven to be more durable; in this regard, the study reported by Tyrka et al. (20) determined that stressful events can lead to epigenetic changes that remain in leukocytes and this methylation could alter the expression of GR and contribute to the imbalance in homeostasis of the HPA axis. According to the above, methylation specifies whether it could mean a reduction in protein levels and could affect GR expression in target cells.

The results of the present report suggest that the analysis of blood methylation is a potential biomarker due to its easy access and availability at any moment of pregnancy. A marker that distinguishes women with a high or low risk can aid in the selection of patients and offer supervision with a better delivery of the available resources. One of the main challenges within the area of preventive medicine is to find biomarkers capable of identifying the onset of a disease, especially in a simple non-invasive manner.

The importance of diagnostic and prognosis PE data is due mainly to its close relationship with maternal mortality in our country and the world (156 maternal accumulated deaths in week 11 of 2018 in Mexico, 21.2% associated with pregnancy hypertensive disorders) (21). The possibility of finding potential biomarkers as well as contributing to the comprehension of the articulated pathophysiological mechanisms makes this relevant in the medical arena. An early therapeutic intervention with diverse targets, starting with the justification of using acetylsalicylic acid or low-molecular-weight heparin, as the ACOG guides suggest, and psychotherapeutical interventions for containment and stress management are some examples (18).

In spite of efforts from public health specialists to decrease its impact, in a study of 830 puerperal females, 13% of those explored were diagnosed with PE, suggesting that the problem is prevalent. Within this group, 17% had EOPE, which worsens the matter because according to the American Heart Association, PE before 34 weeks of gestation relates to a higher cardiovascular risk factor than LOPE (18).

In conclusion, this study found a differential methylation in the GR NR3C1 and its co-chaperone HSP90AA1 between preeclamptic and normotensive women, with a possible regulatory role in the response to stress in pregnancy and a possible physiopathological mechanism in PE. These findings contribute to the strengthening of the causal network paradigm previously described by other authors (6, 8, 22–24). Moreover, they support the relation between PE and an unfavorable psychosocial environment, an imbalance in allostatic factors that contribute to a limited placenta formation that can even be present before PE occurence.

## Strenghts and Limitations

One of the strengths of this study is the design and its strict control of the inclusion criteria. Unfortunately, due to these criteria, a large number of women had to be excluded, resulting in a small sample that finally had an impact on the confidence intervals. Considering the relevance of our findings, studies involving a larger sample are suggested for the future, as well as the use of additional technical techniques that show protein expression or gene sequencing.

## Data Availability Statement

All datasets generated for this study are included in the article/Supplementary Material.

## Ethics Statement

The studies involving human participants were reviewed and approved by Commitee of Ethics in Research of the General Hospital of Durango with registration number 456/015. The patients/participants provided their written informed consent to participate in this study.

## Author Contributions

QT-S performed experimental and statistical work, wrote the manuscript. MR-R performed the methylation analyses. AS-Á participated in the recruitment of cases. RP-M participated in the interpretation of the results, their discussion and wrote the manuscript. YM-L and JS-M participated in the design and performed of the study, wrote the manuscript and approval of the final version.

## Conflict of Interest

The authors declare that the research was conducted in the absence of any commercial or financial relationships that could be construed as a potential conflict of interest.
