# Supplementary material for: Differential Methylation in Promoter Regions of the Genes NR3C1 and HSP90AA1, Involved in the Regulation, and Bioavailability of Cortisol in Leukocytes of Women With Preeclampsia
Source: Front Med (Lausanne). 2020 Jun 16;7:206. doi: 10.3389/fmed.2020.00206 (PMC7326011; doi:10.3389/fmed.2020.00206)
Supplement: Supplementary file 1 [file Data_Sheet_1.PDF]

TABLE No.1 SOCIODEMOGRAPHIC CHARACTERISTICS FROM A SAMPLE OF WOMEN DIAGNOSED WITH PREECLAMPSIA AND NORMOTENSIVE

		Cases (n=20)		Controls (n=20)		*p*
Origin						
	Rural	15	75.0%	15	75.0%	
	Urban	5	25.0%	5	25.0%	*1*
Occupation	Seamstress	1	5.0%	0	_	
	Teacher	1	5.0%	0	_	
	Housewife	17	85.0%	19	95.0%	
	Office employee	0	_	1	5.0%	
	Student	1	5.0%	0	_	*0.391*
Marital status						
	Single	3	15.0%	7	35.0%	
	Married	6	30.0%	2	10.0%	
	Domestic partnership	11	55.0%	11	55.0%	*0.87*

TABLE No.2 CLINICAL INFORMATION OBTAINED FROM A SAMPLE OF PRECLAMPTIC WOMEN VS NORMOTENSIVE

	CASES (n=20)		CONTROLS (n=20)		*p*
Maternal age*	28.45	± 7.6	28.5	± 7.8	*0.969*
Gestational age**	31	(28 - 33)	39.3	(38 - 40)	*0.001*
SBP**	160	(150-172)	110	(92-120)	*0.001*
DBP**	105	(91-110)	67.5	(60-77)	*0.001*
MBP**	122	(112-127)	81.6	(73-89)	*0.001*
	n	%	n	%	
Sex of baby					
Male	12	60.0%	13	65.0%	
Female	8	40.0%	7	35.0%	*0.114*
End of pregnancy					
Caesarean section	19	95%	4	20.0%	
Vaginal delivery	1	5%	16	80.0%	*0.001*
First pregnancy	9	45.0%	3	15.0%	
More than one pregnancy	11	55.0%	17	85.0%	*0.011*

SBP= Systolic blood pressure, DBP= Diastolic blood pressure, MBP= Maximum blood pressure

TABLE No.3 DIFFERENTIAL METHYLATION IN THE CpG ISLANDS OF GENE REGULATORS OF HYPOTHALAMIC-PITUITARY- ADRENAL AXIS

GENE	Chromosomal location	CpG Island	Mean of % of methylation (q25 – q75)				*P*
			Women with preclampsia		Women without preeclampsia		
NR3C1	5q31.3	111914	89.2	(73.3 – 97.2)	22.1	(0.27 – 50)	*0.0003**
CYP11A1	6p21.31	112418	44.0	(0.8 – 88.4)	3.7	(0.42 – 68.7)	*0.377*
TEAD3	16q22.1	105382	90.0	(50 – 97.2)	51.8	(1.72 – 81.6)	*0.077*
HSD11B2	15q24.1	104679	60.7	(1.46 – 95.4)	80.8	(1.73 – 99.90)	*0.446*
HSP90AA1	14q32.31	104327	70.2	(0.40 – 79.4)	1.3	(0.05 – 8.4)	*0.068*
CRHBP	5q13.3	111616	50.0	(33.12 – 64.1)	40.0	(0.52 – 95.2)	*0.579*

N= 20 cases and 20 controls

TABLE No.4 CORRELATION ANALYSIS BETWEEN GENE METHYLATION AND BLOOD PRESSURE

GENE	SBP Coefficient of correlation	SBP *p*	DBP Coefficient of correlation	DBP *p*
NR3C1	0.547	*0.002**	0.562	*0.001**
CYP11A1	0.202	*0.27*	0.19	*0.305*
TEAD3	0.469	*0.05**	0.551	*0.018**
HSD11B2	-0.058	*0.755*	0.071	*0.706*
HSP90AA1	0.379	*0.052*	0.386	*0.046**
CRHBP	0.131	*0.525*	0.222	*0.275*

[1] The information was analyzed using the Spearman’s rank correlation

N= 40 * Statistical significance ≤ 0.05

SBP= Systolic blood preassure, DBP= Diastolic blood preassure

TABLE No.5 HIPERMETHYLATION FREQUENCY[1] IN THE GENES OF STUDY. DIFFERENCES BETWEEN CASES AND CONTROLS

	Women with preeclampsia	Women without preeclampsia	x^2	*P*	OR	IC 95%
NR3C1	46.7%	6.7%	6.13	0.013*	12.25	(1.27 – 118.37)
CYP11A1	56.2%	40.0%	0.819	0.366	1.92	(0.46 – 8.05)
HSD11B2	50.0%	60.0%	0.313	0.576	0.66	(0.16 – 2.77)
HSP90AA1	64.3%	15.4%	6.6	0.01*	9.9	(1.54 – 63.69)
CRHBP	15.4%	30.8%	0.352	0.867	0.4	(0.06 – 2.77)

[1] The cut point was taken as appointed by the cut off finder (18)

Statistical significance $p \leq 0.05$

Supplementary Chart No.1

CpG islands analyzed with the system EpiTect Methyl II qPCR Primer Assay Kit (Qiagen)

GENE OF STUDY	CpG ISLAND	GENOMIC COORDINATES
NR3C1	112418	Chr 6: 34 464 056 – 35 466 269
CYP11A1	105382	Chr 16: 67 464 388 – 67 465 462
TEAD3	104679	Chr 15: 74 658 038 – 74 658 574
HSD11B2	104327	Chr 14: 102 552 721 – 102 554 068
HSP90AA1	111616	Chr 5: 76 249 435 – 76 250 528
CRHBP	111914	Chr 5: 142 782 071 – 142 785 071
